# *N*-Acetyl-5-chloro-3-nitro-l-tyrosine ethyl ester

**DOI:** 10.1107/S1600536812036380

**Published:** 2012-08-31

**Authors:** Teresa T. Mutahi, Benson J. Edagwa, Frank R. Fronczek, Rao M. Uppu

**Affiliations:** a Department of Environmental Toxicology and the Health Research Center, Southern University and A&M College, Baton Rouge, LA 70813, USA; b Department of Chemistry, Louisiana State University, Baton Rouge, LA 70803-1804, USA

## Abstract

The title compound, C_13_H_15_ClN_2_O_6_, was synthesized by hypochlorous acid-mediated chlorination of *N*-acetyl-3-nitro-l-tyrosine ethyl ester. The OH group forms an intra­molecular O—H⋯O hydrogen bond to the nitro group and the N—H group forms an inter­molecular N—H⋯O hydrogen bonds to an amide O atom, linking the mol­ecules into chains along [100]. The crystal studied was a non-merohedral twin, with a 0.907 (4):0.093 (4) domain ratio.

## Related literature

For background to per­oxy­nitrite and its reactions with amino acids, see: Alvarez *et al.* (1999 ▶); Beckman (2009 ▶); Ceriello (2002 ▶); Crow (1999 ▶); Dahaoui *et al.* (1999 ▶); Darwish *et al.* 2007 ▶; Janik *et al.* (2007 ▶, 2008 ▶); Koszelak & van der Helm (1981 ▶); Pieret *et al.* (1972 ▶); Pitt & Spickett (2008 ▶); Soriano-García (1993) ▶; Stout *et al.* (2000 ▶); Uppu & Pryor (1999 ▶); Uppu *et al.* (1996 ▶); Whiteman & Halliwell (1999 ▶); Winter­bourn (2002 ▶).
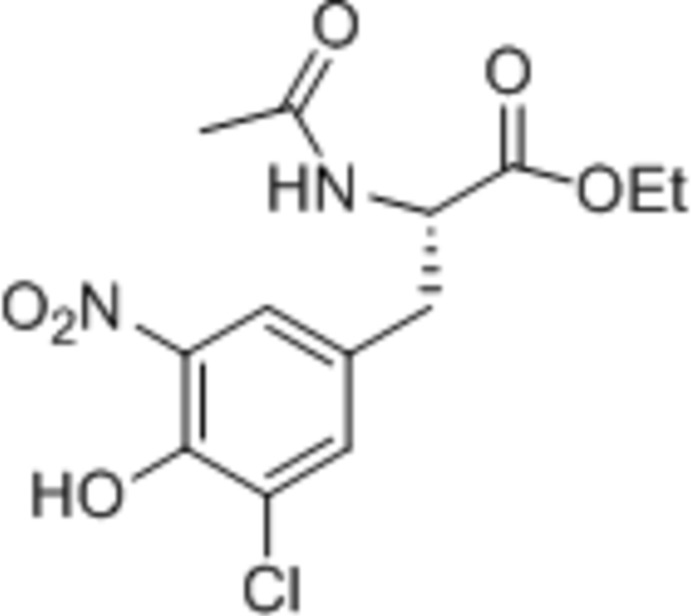


## Experimental

### 

#### Crystal data

C_13_H_15_ClN_2_O_6_
*M* *_r_* = 330.72
Monoclinic, 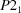

*a* = 5.1513 (4) Å
*b* = 10.6761 (9) Å
*c* = 13.2849 (8) Å
β = 93.689 (4)°
*V* = 729.10 (9) Å^3^
*Z* = 2
Cu *K*α radiation
μ = 2.63 mm^−1^
*T* = 90 K
0.34 × 0.11 × 0.03 mm

#### Data collection

Bruker Kappa APEXII DUO area-detector diffractometer
Absorption correction: multi-scan (*TWINABS*; Sheldrick, 2002 ▶) *T*_min_ = 0.468, *T*_max_ = 0.925
7589 measured reflections
2307 independent reflections
2299 reflections with *I* > 2σ(*I*)
*R*_int_ = 0.058

#### Refinement

*R*[*F* ^2^ > 2σ(*F* ^2^)] = 0.034
*wR*(*F* ^2^) = 0.091
*S* = 1.07
2307 reflections
208 parameters
2 restraints
H atoms treated by a mixture of independent and constrained refinement
Δρ_max_ = 0.32 e Å^−3^
Δρ_min_ = −0.20 e Å^−3^
Absolute structure: Flack (1983 ▶), 961 Friedel pairs
Flack parameter: 0.078 (17)

### 

Data collection: *APEX2* (Bruker, 2006 ▶); cell refinement: *SAINT* (Bruker, 2006 ▶); data reduction: *SAINT*; program(s) used to solve structure: *SHELXS97* (Sheldrick, 2008 ▶); program(s) used to refine structure: *SHELXL97* (Sheldrick, 2008 ▶); molecular graphics: *ORTEP-3 for Windows* (Farrugia, 1997 ▶); software used to prepare material for publication: *SHELXTL* (Sheldrick, 2008 ▶).

## Supplementary Material

Crystal structure: contains datablock(s) global, I. DOI: 10.1107/S1600536812036380/hb6933sup1.cif

Structure factors: contains datablock(s) I. DOI: 10.1107/S1600536812036380/hb6933Isup2.hkl

Additional supplementary materials:  crystallographic information; 3D view; checkCIF report

## Figures and Tables

**Table 1:** Hydrogen-bond geometry (Å, °)

*D*—H⋯*A*	*D*—H	H⋯*A*	*D*⋯*A*	*D*—H⋯*A*
O1—H1*O*⋯O2	0.96 (4)	1.63 (4)	2.570 (3)	168 (3)
N2—H2*N*⋯O6^i^	0.82 (2)	2.23 (2)	2.999 (3)	156 (3)

Symmetry code: (i) 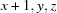.

**Fig. 1.**
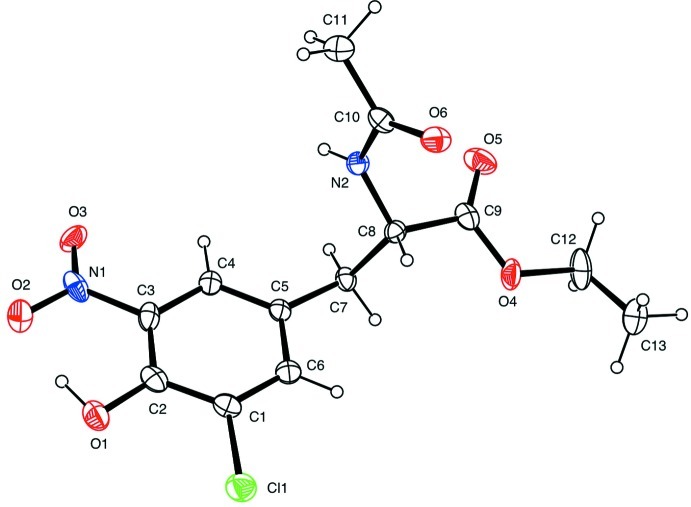
Ellipsoids at the 50% level, with H atoms having arbitrary radius.

**Fig. 2.**
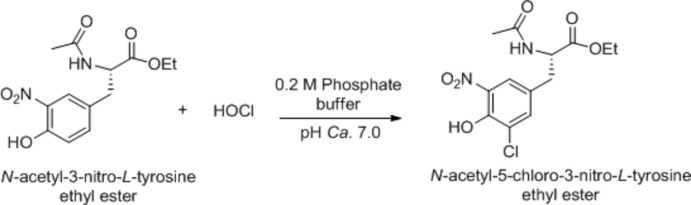
Chlorination of N-acetyl-3-nitro-L-tyrosine ethyl ester by hypochlorite/hypochlorous acid

**Fig. 3.**
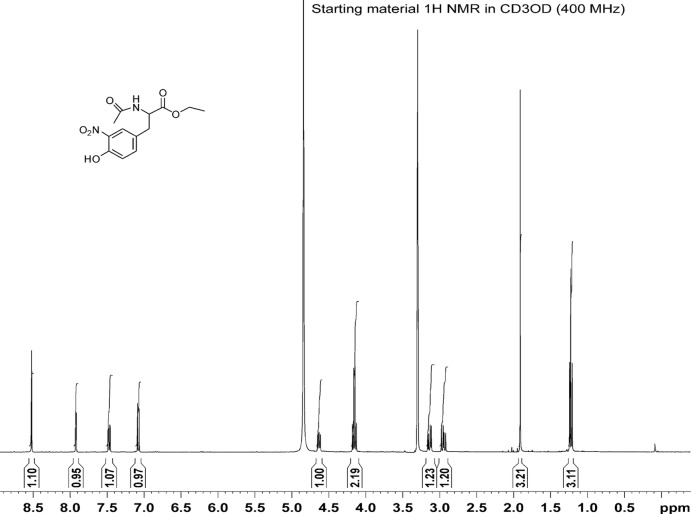
1H-NMR spectrum of N-acetyl-3-nitro-L-tyrosine ethyl ester dissolved in CD3OD and analyzed on a Bruker AV-400-liquid spectrometer.

**Fig. 4.**
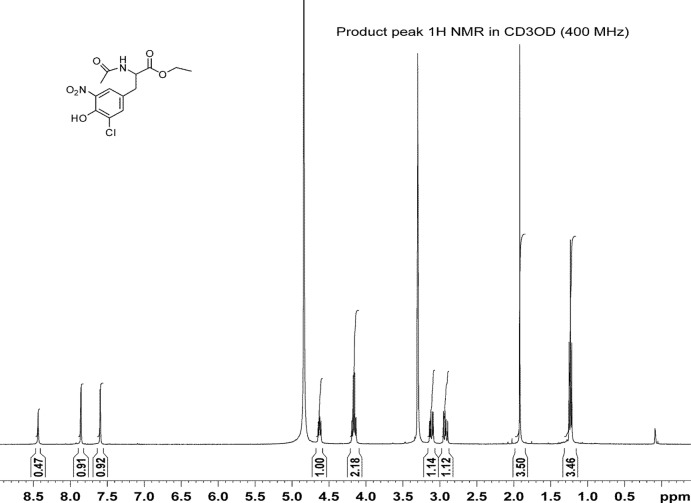
1H-NMR spectrum of N-acetyl-5-chloro-3-nitro-L-tyrosine ethyl ester in CD3OD and analyzed on a Bruker AV-400-liquid spectrometer.

## supplementary crystallographic information

### Comment

Peroxynitrite (PN), an oxidant formed during the down-regulation of nitric oxide (^.^NO) (Uppu & Pryor, 1999), is known to cause oxidation of both free- and protein-bound amino acids (AAs) (Alverez *et al.*, 1999; Beckman 2009; Uppu *et al.*, 1996). The reactivity of PN towards AAs in proteins can be accounted for by the side chains of constituent AAs in particular those present in cysteine, methionine, tyrosine, tryptophan, and histidine. Among the various AAs with reactive side chains, the oxidation of Tyr by PN results in the formation of a characteristic nitro product, 3-nitroTyr (3-NO_2_Tyr) (Beckman, 2009; Ceriello, 2002; Crow, 1999; Darwish *et al.*, 2007) which is often used as a marker of PN formation *in vivo*. Hypochlorous acid (HOCl) is another oxidant that can also be formed at sites of inflammation, catalyzed by the enzyme myeloperoxidase. Like PN, HOCl is mostly reactive towards the side chains of cysteine, methionine, tyrosine, tryptophan, and histidine and cause posttranslational modifications of proteins resulting in chlorinated products. 3-Chloro-*L*-tyrosine is one the products that has been well characterized and used as a biomarker of HOCl formation *in vivo* (Crow, 1999; Pitt & Spickett, 2008; Winterbourn, 2002). Now, a question that follows naturally but never addressed in detail is what happens when HOCl and PN are produced in the same biological *milieu* and react with AA side chains in proteins. The significance of these combined oxidations on the issue of biomarker validation could be truly overwhelming given the report by Whiteman and Halliwell (1999) wherein it was shown that the 3-NO_2_Tyr was in fact lost to some unknown product(s) following oxidation with HOCl. Another important consequence could be that we need additional biomarkers and their validation.

Herein, we report the synthesis and characterization of the oxidation product of HOCl reaction with *N*-acetyl-3-nitro-*L*-tyrosine ethyl ester (NANTEE), a model for protein-bound 3-NO_2_Tyr. When HOCl was a limiting reagent (hypochlorite/HOCl < NANTEE), the major product was found to be *N*-acetyl-5-chloro-3-nitro-*L*-tyrosine ethyl ester (NACNTEE). This product was purified by reversed phase (RP) high-performance liquid chromatography (HPLC). Its identification was based on single-crystal X-ray crystallographic analysis (Fig. 1) and ^1^H-NMR assignments (Figs. 2–4).

The structure is shown in Fig. 1. The absolute configuration at the asymmetric center C8 is S, in agreement with the known configuration of the starting material. Molecular geometry is normal, except for the nitro group, which has slightly long C3—N1 distance, 1.473 (4) Å and asymmetric N—O distances, N1—O2 1.249 (3) and N1—O3 1.169 (3) Å. The shape of the N1 ellipsoid is somewhat peculiar, while ellipsoids for other atoms in the molecule appear normal. The two C—C—N angles at the nitro-substituted C atom C3 also differ by 3.5 (3)°. These features suggest the possibility of a slight disorder involving rotation of the phenyl group, such that the Cl atom nearly superimposes upon N1 a small fraction of the time. This would lead to a slightly misplaced refined N1 position and account for the observed irregularities.

The nitro group lies nearly in the phenyl plane, with O2—N1—C3—C2 torsion angle 2.6 (3)°, and it accepts an intramolecular hydrogen bond from the OH group, having O1···O2 distance 2.570 (3) Å. The tyrosine *N*-acetyl NH group donates an intermolecular hydrogen bond to O6 (at *x* + 1, *y*, *z*), forming chains in the [1 0 0] direction.

### Experimental

Chemicals and solvents used in the preparation and recrystallization of NACNTEE were obtained as follows: NANTEE, potassium phosphate monobasic, sodium phosphate dibasic, sodium hydroxide, sodium hypochlorite (chlorine content: *ca*. 5%), CD_3_OD from Sigma (St. Louis, MO); formic acid (88%) from Fishers chemicals (Fair Lawn, NJ); ammonium hydroxide (28–30%)from VWR (Goshen Parkway, PA); HPLC grade methanol from EMD Chemicals (Gibbstown, NJ). Water with resistance of 18 megaohms/cm or higher was used.

Oxidation of NANTEE was performed by reacting equimollar concentrations of NANTEE with hypochlorite/HOCl. Briefly, NANTEE (8.5 mg) was dissolved in 2.8 m*L* of 0.2 *M* phosphate buffer, pH 7.0 to make a 10 m*M* NANTEE solution. A solution of 56 µ*L* of hypochlorite (stock solution) was added drop-wise to the 10 m*M* NANTEE solution while stirring. Aliquots (200 µ*L* each) of the reaction mixture were analyzed by reversed phase HPLC using Supleco LC18 column (150 *x* 4.6 mm, particle size: 5µ) and an isocratic mobile phase consisting of 0.05*M* ammonium formate buffer solution (50%) and methanol (50%) at pH of 3.93 and a flow rate of 1 m*L*/min. The absorbance was set at 410 nm. The HPLC system used in this research was a Lab Alliance series II/III liquid chromatography equipped with Lab Alliance model 500 UV-Vis detector and Peak Simple 329 chromatography data system. The peaks corresponding to pure NANTEE and the product were collected and concentrated. The amorphous powder was recrystallized from methanol to give yellow needles of NACNTEE. For ^1^H-NMR spectrum, both NANTEE and NACNTEE were dissolved in CD_3_OD and analyzed on a Bruker AV-400-liquid spectrometer. The ^1^H-NMR data are reported in ppm downfield from TMS as an internal standard.

*N*-acetyl-3-nitro-*L*-tyrosine ethyl ester (Fig. 3): ^1^H-NMR (400 MHz, CD_3_OD): δ 1.23 (t, J = 7.1 Hz, 3H), 1.91 (s, 3H), 2.95 (dd, J = 14.0, 8.7 Hz, 1H), 3.14 (dd, J = 14.0, 5.8 Hz, 1H), 4.15 (q, J = 7.1 Hz, 2H), 4.63 (dd, J = 8.8, 5.8 Hz, 1H), 7.08 (d, J = 8.6 Hz, 1H), 7.47 (dd, J = 8.6, 2.2 Hz, 1H), 7.92 (d, J = 2.1 Hz, 1H), 8.52 (s, 1H).

*N*-acetyl-5-chloro-3-nitro-*L*-tyrosine ethyl ester (Fig. 4): ^1^H-NMR (400 MHz, CD_3_OD): δ 1.23 (t, J = 7.1 Hz, 3H), 1.92 (s, 3H), 2.92 (dd, J = 14.1, 8.7 Hz, 1H), 3.12 (dd, J = 14.1, 5.8 Hz, 1H), 4.16 (q, J = 7.0 Hz, 2H), 4.63 (dd, J = 8.6, 5.8 Hz, 1H), 7.60 (d, J = 2.1 Hz, 1H), 7.86 (d, J = 2.1 Hz, 1H), 8.43 (s, 1H) (Fig. 4). The chemical shifts derived from the proton NMR spectrum of the product are consistent with the structure of *N*-acetyl-5-chloro-3-nitro-*L*-tyrosine ethyl ester, specifically, the doublet at 7.08 ppm corresponding to the proton at the *ortho* position of the OH group on the aromatic ring in the starting material disappears in the product due to chloride substitution.

### Refinement

H atoms on C were placed in idealized positions, with C—H distances 0.95–1.00 Å. A torsional parameter was refined for each methyl group. N—H and hydroxy H atom positions were refined. *U*_iso_ for H were assigned as 1.2 times *U*_eq_ of the attached atoms (1.5 for methyl and OH).

### Crystal data

**Table:**

C_13_H_15_ClN_2_O_6_	*F*(000) = 344
*M**_r_* = 330.72	*D*_x_ = 1.506 Mg m^−^^3^
Monoclinic, *P*2_1_	Cu *K*α radiation, λ = 1.54184 Å
Hall symbol: P 2yb	Cell parameters from 1900 reflections
*a* = 5.1513 (4) Å	θ = 7.9–67.6°
*b* = 10.6761 (9) Å	µ = 2.63 mm^−^^1^
*c* = 13.2849 (8) Å	*T* = 90 K
β = 93.689 (4)°	Lath, yellow
*V* = 729.10 (9) Å^3^	0.34 × 0.11 × 0.03 mm
*Z* = 2	

### Data collection

**Table:**

Bruker Kappa APEXII DUO area-detector diffractometer	2307 independent reflections
Radiation source: IµS microfocus	2299 reflections with *I* > 2σ(*I*)
QUAZAR multilayer optics monochromator	*R*_int_ = 0.058
φ and ω scans	θ_max_ = 68.2°, θ_min_ = 6.7°
Absorption correction: multi-scan (TWINABS; Sheldrick, 2002)	*h* = −6→6
*T*_min_ = 0.468, *T*_max_ = 0.925	*k* = −12→12
7589 measured reflections	*l* = −15→15

### Refinement

**Table:**

Refinement on *F*^2^	Secondary atom site location: difference Fourier map
Least-squares matrix: full	Hydrogen site location: inferred from neighbouring sites
*R*[*F*^2^ > 2σ(*F*^2^)] = 0.034	H atoms treated by a mixture of independent and constrained refinement
*wR*(*F*^2^) = 0.091	*w* = 1/[σ^2^(*F*_o_^2^) + (0.029*P*)^2^ + 0.4076*P*] where *P* = (*F*_o_^2^ + 2*F*_c_^2^)/3
*S* = 1.07	(Δ/σ)_max_ < 0.001
2307 reflections	Δρ_max_ = 0.32 e Å^−^^3^
208 parameters	Δρ_min_ = −0.20 e Å^−^^3^
2 restraints	Absolute structure: Flack (1983), 961 Friedel pairs
Primary atom site location: structure-invariant direct methods	Flack parameter: 0.078 (17)

### Special details

**Table:**

Geometry. All e.s.d.'s (except the e.s.d. in the dihedral angle between two l.s. planes) are estimated using the full covariance matrix. The cell e.s.d.'s are taken into account individually in the estimation of e.s.d.'s in distances, angles and torsion angles; correlations between e.s.d.'s in cell parameters are only used when they are defined by crystal symmetry. An approximate (isotropic) treatment of cell e.s.d.'s is used for estimating e.s.d.'s involving l.s. planes.
Refinement. The crystal not single, and was treated as a nonmerohedral twin by rotation of 4.6 degrees about reciprocal axis 0.070 1.000 - 0.042 and real axis 0.300 1.000 - 0.019 The twin law is: (0.991, 0.000, -0.032, 0.006, 1.000, 0.014, 0.215, -0.021, 1.002)The structure was refined *versus*. TWIN5 data, yielding BASF=0.093 (4).

### Fractional atomic coordinates and isotropic or equivalent isotropic displacement parameters (Å^2^)

**Table:**

	*x*	*y*	*z*	*U*_iso_*/*U*_eq_	
Cl1	0.24004 (11)	0.34934 (7)	0.34487 (4)	0.02854 (18)	
O1	0.6456 (4)	0.53157 (19)	0.31400 (14)	0.0267 (4)	
H1O	0.792 (8)	0.585 (4)	0.330 (3)	0.040*	
O2	1.0367 (4)	0.66367 (19)	0.38081 (15)	0.0331 (5)	
O3	1.1791 (4)	0.65600 (18)	0.53520 (16)	0.0286 (5)	
O4	0.2148 (4)	0.23412 (19)	0.84645 (13)	0.0267 (4)	
O5	0.4182 (4)	0.37138 (19)	0.95279 (13)	0.0316 (5)	
O6	0.0002 (3)	0.58322 (18)	0.81904 (14)	0.0245 (4)	
N1	1.0294 (4)	0.6262 (2)	0.46967 (16)	0.0248 (5)	
N2	0.4220 (4)	0.5472 (2)	0.79647 (15)	0.0175 (4)	
H2N	0.568 (4)	0.579 (3)	0.797 (2)	0.021*	
C1	0.4584 (5)	0.4103 (3)	0.43817 (19)	0.0220 (6)	
C2	0.6440 (5)	0.4968 (2)	0.41110 (19)	0.0215 (5)	
C3	0.8183 (5)	0.5385 (2)	0.4902 (2)	0.0216 (5)	
C4	0.8039 (5)	0.4991 (2)	0.58960 (18)	0.0175 (5)	
H4	0.9214	0.5319	0.6410	0.021*	
C5	0.6194 (4)	0.4125 (2)	0.61350 (18)	0.0163 (5)	
C6	0.4450 (5)	0.3693 (2)	0.53587 (18)	0.0190 (5)	
H6	0.3147	0.3105	0.5510	0.023*	
C7	0.6044 (4)	0.3609 (2)	0.71852 (17)	0.0175 (5)	
H7A	0.7695	0.3792	0.7581	0.021*	
H7B	0.5847	0.2688	0.7146	0.021*	
C8	0.3772 (4)	0.4158 (2)	0.77388 (18)	0.0166 (5)	
H8	0.2149	0.4085	0.7287	0.020*	
C9	0.3412 (4)	0.3398 (3)	0.86964 (18)	0.0202 (5)	
C10	0.2262 (5)	0.6225 (2)	0.81958 (18)	0.0194 (5)	
C11	0.2946 (6)	0.7547 (3)	0.8436 (2)	0.0266 (6)	
H11A	0.2472	0.8076	0.7849	0.040*	
H11B	0.4822	0.7612	0.8605	0.040*	
H11C	0.1998	0.7827	0.9011	0.040*	
C12	0.1647 (7)	0.1488 (3)	0.9296 (2)	0.0381 (7)	
H12A	0.1447	0.1969	0.9923	0.046*	
H12B	0.3119	0.0899	0.9414	0.046*	
C13	−0.0777 (7)	0.0789 (3)	0.9011 (3)	0.0415 (8)	
H13A	−0.2226	0.1379	0.8908	0.062*	
H13B	−0.1142	0.0202	0.9551	0.062*	
H13C	−0.0565	0.0323	0.8386	0.062*	

### Atomic displacement parameters (Å^2^)

**Table:**

	*U*^11^	*U*^22^	*U*^33^	*U*^12^	*U*^13^	*U*^23^
Cl1	0.0268 (3)	0.0336 (3)	0.0250 (3)	−0.0030 (3)	0.0000 (2)	−0.0031 (3)
O1	0.0312 (11)	0.0286 (10)	0.0207 (9)	0.0023 (9)	0.0058 (8)	0.0033 (7)
O2	0.0380 (12)	0.0302 (11)	0.0321 (11)	−0.0054 (9)	0.0103 (9)	0.0044 (8)
O3	0.0221 (10)	0.0241 (10)	0.0401 (12)	−0.0107 (8)	0.0066 (9)	−0.0074 (8)
O4	0.0307 (10)	0.0265 (10)	0.0234 (9)	−0.0079 (8)	0.0060 (7)	0.0089 (8)
O5	0.0478 (12)	0.0295 (11)	0.0175 (9)	0.0062 (10)	0.0015 (8)	−0.0004 (8)
O6	0.0149 (9)	0.0286 (10)	0.0303 (10)	0.0014 (8)	0.0026 (7)	−0.0090 (8)
N1	0.0261 (12)	0.0275 (12)	0.0221 (12)	0.0139 (10)	0.0115 (10)	0.0057 (9)
N2	0.0139 (10)	0.0191 (10)	0.0196 (10)	−0.0005 (8)	0.0027 (8)	−0.0019 (8)
C1	0.0225 (12)	0.0236 (13)	0.0200 (12)	0.0036 (11)	0.0024 (10)	−0.0037 (10)
C2	0.0221 (12)	0.0219 (12)	0.0209 (13)	0.0097 (10)	0.0058 (9)	−0.0001 (10)
C3	0.0183 (13)	0.0170 (12)	0.0306 (14)	0.0038 (10)	0.0113 (10)	0.0040 (10)
C4	0.0160 (12)	0.0157 (11)	0.0210 (12)	0.0033 (9)	0.0030 (9)	0.0010 (9)
C5	0.0148 (11)	0.0151 (11)	0.0196 (12)	0.0035 (10)	0.0051 (9)	−0.0009 (9)
C6	0.0200 (11)	0.0150 (12)	0.0223 (11)	0.0043 (10)	0.0034 (8)	−0.0009 (9)
C7	0.0158 (10)	0.0185 (12)	0.0188 (11)	0.0015 (10)	0.0049 (8)	0.0011 (10)
C8	0.0150 (11)	0.0163 (11)	0.0188 (12)	−0.0002 (10)	0.0018 (9)	−0.0004 (9)
C9	0.0178 (11)	0.0210 (12)	0.0225 (12)	0.0088 (11)	0.0063 (9)	0.0025 (11)
C10	0.0181 (13)	0.0257 (13)	0.0143 (11)	0.0049 (10)	−0.0001 (9)	0.0001 (9)
C11	0.0296 (14)	0.0239 (14)	0.0264 (13)	0.0019 (12)	0.0012 (10)	−0.0039 (11)
C12	0.0403 (18)	0.0433 (18)	0.0317 (16)	−0.0048 (15)	0.0093 (13)	0.0229 (14)
C13	0.050 (2)	0.0364 (17)	0.0398 (17)	−0.0137 (16)	0.0147 (14)	0.0072 (15)

### Geometric parameters (Å, º)

**Table:**

Cl1—C1	1.745 (3)	C5—C6	1.402 (3)
O1—C2	1.343 (3)	C5—C7	1.507 (3)
O1—H1O	0.96 (4)	C6—H6	0.9500
O2—N1	1.249 (3)	C7—C8	1.538 (3)
O3—N1	1.169 (3)	C7—H7A	0.9900
O4—C9	1.329 (4)	C7—H7B	0.9900
O4—C12	1.467 (3)	C8—C9	1.530 (3)
O5—C9	1.198 (3)	C8—H8	1.0000
O6—C10	1.237 (3)	C10—C11	1.485 (4)
N1—C3	1.473 (4)	C11—H11A	0.9800
N2—C10	1.341 (3)	C11—H11B	0.9800
N2—C8	1.449 (3)	C11—H11C	0.9800
N2—H2N	0.823 (18)	C12—C13	1.483 (5)
C1—C6	1.375 (4)	C12—H12A	0.9900
C1—C2	1.393 (4)	C12—H12B	0.9900
C2—C3	1.409 (4)	C13—H13A	0.9800
C3—C4	1.393 (4)	C13—H13B	0.9800
C4—C5	1.377 (4)	C13—H13C	0.9800
C4—H4	0.9500		
			
C2—O1—H1O	91 (2)	H7A—C7—H7B	107.8
C9—O4—C12	117.4 (2)	N2—C8—C9	111.5 (2)
O3—N1—O2	123.9 (2)	N2—C8—C7	110.6 (2)
O3—N1—C3	119.6 (2)	C9—C8—C7	109.38 (19)
O2—N1—C3	116.5 (2)	N2—C8—H8	108.4
C10—N2—C8	121.0 (2)	C9—C8—H8	108.4
C10—N2—H2N	117 (2)	C7—C8—H8	108.4
C8—N2—H2N	122 (2)	O5—C9—O4	125.5 (2)
C6—C1—C2	122.1 (2)	O5—C9—C8	124.5 (3)
C6—C1—Cl1	118.9 (2)	O4—C9—C8	110.0 (2)
C2—C1—Cl1	119.0 (2)	O6—C10—N2	121.1 (2)
O1—C2—C1	118.5 (2)	O6—C10—C11	122.3 (2)
O1—C2—C3	125.9 (2)	N2—C10—C11	116.6 (2)
C1—C2—C3	115.6 (2)	C10—C11—H11A	109.5
C4—C3—C2	122.8 (2)	C10—C11—H11B	109.5
C4—C3—N1	116.9 (2)	H11A—C11—H11B	109.5
C2—C3—N1	120.3 (2)	C10—C11—H11C	109.5
C5—C4—C3	120.1 (2)	H11A—C11—H11C	109.5
C5—C4—H4	120.0	H11B—C11—H11C	109.5
C3—C4—H4	120.0	O4—C12—C13	107.8 (2)
C4—C5—C6	118.1 (2)	O4—C12—H12A	110.1
C4—C5—C7	122.4 (2)	C13—C12—H12A	110.1
C6—C5—C7	119.5 (2)	O4—C12—H12B	110.1
C1—C6—C5	121.4 (2)	C13—C12—H12B	110.1
C1—C6—H6	119.3	H12A—C12—H12B	108.5
C5—C6—H6	119.3	C12—C13—H13A	109.5
C5—C7—C8	112.9 (2)	C12—C13—H13B	109.5
C5—C7—H7A	109.0	H13A—C13—H13B	109.5
C8—C7—H7A	109.0	C12—C13—H13C	109.5
C5—C7—H7B	109.0	H13A—C13—H13C	109.5
C8—C7—H7B	109.0	H13B—C13—H13C	109.5
			
C6—C1—C2—O1	179.6 (2)	C4—C5—C6—C1	0.9 (3)
Cl1—C1—C2—O1	0.9 (3)	C7—C5—C6—C1	−177.4 (2)
C6—C1—C2—C3	0.6 (4)	C4—C5—C7—C8	105.2 (3)
Cl1—C1—C2—C3	−178.02 (18)	C6—C5—C7—C8	−76.6 (3)
O1—C2—C3—C4	179.5 (2)	C10—N2—C8—C9	−75.9 (3)
C1—C2—C3—C4	−1.6 (4)	C10—N2—C8—C7	162.1 (2)
O1—C2—C3—N1	−1.3 (4)	C5—C7—C8—N2	−68.5 (3)
C1—C2—C3—N1	177.5 (2)	C5—C7—C8—C9	168.3 (2)
O3—N1—C3—C4	2.3 (4)	C12—O4—C9—O5	0.1 (4)
O2—N1—C3—C4	−178.1 (2)	C12—O4—C9—C8	179.6 (2)
O3—N1—C3—C2	−176.9 (2)	N2—C8—C9—O5	−22.1 (3)
O2—N1—C3—C2	2.6 (3)	C7—C8—C9—O5	100.6 (3)
C2—C3—C4—C5	2.3 (4)	N2—C8—C9—O4	158.4 (2)
N1—C3—C4—C5	−176.9 (2)	C7—C8—C9—O4	−78.9 (2)
C3—C4—C5—C6	−1.9 (3)	C8—N2—C10—O6	−2.4 (4)
C3—C4—C5—C7	176.4 (2)	C8—N2—C10—C11	178.7 (2)
C2—C1—C6—C5	−0.3 (4)	C9—O4—C12—C13	150.8 (3)
Cl1—C1—C6—C5	178.34 (19)		

### Hydrogen-bond geometry (Å, º)

**Table:**

*D*—H···*A*	*D*—H	H···*A*	*D*···*A*	*D*—H···*A*
O1—H1*O*···O2	0.96 (4)	1.63 (4)	2.570 (3)	168 (3)
N2—H2*N*···O6^i^	0.82 (2)	2.23 (2)	2.999 (3)	156 (3)

Symmetry code: (i) *x*+1, *y*, *z*.

## References

[bb1] Alvarez, B., Ferrer-Sueta, G., Freeman, B. A. & Radi, R. (1999). *J. Biol. Chem.* **274**, 842–848. 10.1074/jbc.274.2.842 9873023

[bb2] Beckman, J. S. (2009). *Arch. Biochem. Biophys.* **484**, 114–116. 10.1016/j.abb.2009.03.013 PMC2709800 19393377

[bb3] Bruker (2006). *APEX2* and *SAINT* Bruker AXS Inc., Madison, Wisconsin, USA.

[bb4] Ceriello, A. (2002). *Int. J. Clin. Pract. Suppl.* **129**, 51–58. 12166608

[bb5] Crow, J. P. (1999). *Methods in Enzymology*, Vol. 301, *Nitric Oxide Part C: Biological and Antioxidant Activities*, edited by L. Packer, pp. 151–160. New York: Academic Press.

[bb6] Dahaoui, S., Jelsch, C., Howard, J. A. K. & Lecomte, C. (1999). *Acta Cryst.* B**55**, 226–230. 10.1107/s0108768198012099 10927361

[bb7] Darwish, R. S., Amiridze, N. & Aarabi, B. (2007). *J. Trauma*, **63**, 439–442. 10.1097/TA.0b013e318069178a 17693848

[bb8] Farrugia, L. J. (1997). *J. Appl. Cryst.* **30**, 565.

[bb9] Flack, H. D. (1983). *Acta Cryst.* A**39**, 876–881.

[bb10] Janik, A., Chyra, A. & Stadnicka, K. (2007). *Acta Cryst.* C**63**, o572–o575. 10.1107/S0108270107038413 17917224

[bb11] Janik, A., Jarocha, M. & Stadnicka, K. (2008). *Acta Cryst.* B**64**, 223–229. 10.1107/S010876810800219X 18369294

[bb12] Koszelak, S. N. & van der Helm, D. (1981). *Acta Cryst.* B**37**, 1122–1124.

[bb13] Pieret, A. F., Durant, F., Germain, G. & Koch, M. (1972). *Cryst. Struct. Commun.* **1**, 75–77.

[bb14] Pitt, A. R. & Spickett, C. M. (2008). *Biochem. Soc. Trans.* **36**, 1077–1082. 10.1042/BST0361077 18793192

[bb15] Sheldrick, G. (2002). *TWINABS* University of Göttingen, Germany.

[bb16] Sheldrick, G. M. (2008). *Acta Cryst.* A**64**, 112–122. 10.1107/S0108767307043930 18156677

[bb17] Soriano-García, M. (1993). *Acta Cryst.* C**49**, 96–97.

[bb18] Stout, K. L., Hallock, K. J., Kampf, J. W. & Ramamoorthy, A. (2000). *Acta Cryst.* C**56**, e100. 10.1107/S0108270100001712 15263215

[bb19] Uppu, R. M. & Pryor, W. A. (1999). *J. Am. Chem. Soc.* **121**, 9738–9739.

[bb20] Uppu, R. M., Squadrito, G. L. & Pryor, W. A. (1996). *Arch. Biochem. Biophys.* **327**, 335-343. 10.1006/abbi.1996.0131 8619624

[bb21] Whiteman, M. & Halliwell, B. (1999). *Biochem. Biophys. Res. Commun.* **258**, 168–172. 10.1006/bbrc.1999.0564 10222254

[bb22] Winterbourn, C. C. (2002). *Toxicology*, **181–182**, 223–227. 10.1016/s0300-483x(02)00286-x 12505315

